# 1-Bromo-2-(4-methoxy­phen­oxy)ethane

**DOI:** 10.1107/S1600536810001893

**Published:** 2010-01-23

**Authors:** Lei Shen, Yong-Hong Hu, Wen-Ge Yang, Xiao-Lei Zhao, Jin-Feng Yao

**Affiliations:** a School of Pharmaceutical Sciences, Nanjing University of Technolgy, Xinmofan Road No. 5 Nanjing, Nanjing 210009, People’s Republic of China; b College of Life Science and Pharmaceutical Engineering, Nanjing University of Technolgy, Xinmofan Road No. 5 Nanjing, Nanjing 210009, People’s Republic of China

## Abstract

In the crystal structure of the title compound, C_9_H_11_BrO_2_, mol­ecules are stacked parallel to the *b*-axis direction, forming double layers in which the molecules are arranged head-to-head, with the bromo­methyl groups pointing towards each other.

## Related literature

For background to the use of the title compound as a pharmaceutical inter­mediate, see: Ran *et al.* (2000 ▶). For bond-length data, see: Allen *et al.* (1987 ▶).
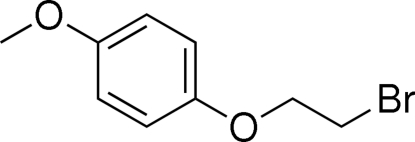


## Experimental

### 

#### Crystal data

C_9_H_11_BrO_2_
*M* *_r_* = 231.09
Monoclinic, 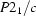

*a* = 21.112 (4) Å
*b* = 5.4180 (11) Å
*c* = 8.3230 (17) Å
β = 94.54 (3)°
*V* = 949.0 (3) Å^3^
*Z* = 4
Mo *K*α radiation
μ = 4.29 mm^−1^
*T* = 293 K
0.20 × 0.10 × 0.10 mm

#### Data collection

Enraf–Nonius CAD-4 diffractometer
Absorption correction: ψ scan (North *et al.*, 1968 ▶) *T*_min_ = 0.481, *T*_max_ = 0.674
1759 measured reflections
1713 independent reflections
1050 reflections with *I* > 2σ(*I*)
*R*_int_ = 0.073
3 standard reflections every 200 reflections  intensity decay: 1%

#### Refinement

*R*[*F* ^2^ > 2σ(*F* ^2^)] = 0.069
*wR*(*F* ^2^) = 0.155
*S* = 1.01
1713 reflections
110 parameters
H-atom parameters constrained
Δρ_max_ = 0.66 e Å^−3^
Δρ_min_ = −0.55 e Å^−3^

### 

Data collection: *CAD-4 Software* (Enraf–Nonius, 1985 ▶); cell refinement: *CAD-4 Software*; data reduction: *XCAD4* (Harms & Wocadlo, 1995 ▶); program(s) used to solve structure: *SHELXS97* (Sheldrick, 2008 ▶); program(s) used to refine structure: *SHELXL97* (Sheldrick, 2008 ▶); molecular graphics: *PLATON* (Spek, 2009 ▶); software used to prepare material for publication: *SHELXTL* (Sheldrick, 2008 ▶).

## Supplementary Material

Crystal structure: contains datablocks I, global. DOI: 10.1107/S1600536810001893/jh2122sup1.cif

Structure factors: contains datablocks I. DOI: 10.1107/S1600536810001893/jh2122Isup2.hkl

Additional supplementary materials:  crystallographic information; 3D view; checkCIF report

## Figures and Tables

**Fig. 1.**
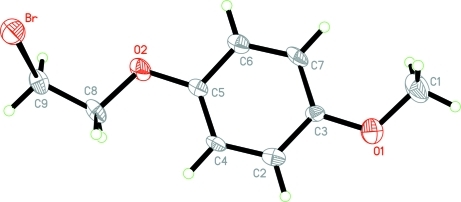
The molecular structure of (I), with the atom-numbering scheme. Displacement ellipsoids are drawn at the 30% probability level.

**Fig. 2.**
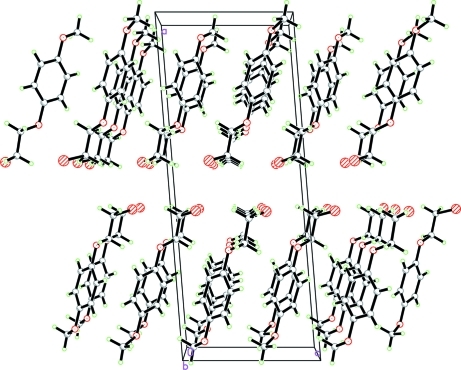
A packing diagram of (I).

## supplementary crystallographic information

### Comment

The tile compound, (I), contains halogen and methoxy groups, which can react with differen groups to prepare various functional organic compounds as a fine organic intermediate (Ran *et al.*, 2000). we report herein its crystal structure.

In the molecule of the tile compound (Fig.1), the bond lengths (Allen *et al.*, 1987) and angles are within normal ranges. The O1 and O2 atoms (Table 1) lie in the benzene ring plane. No intramolecular hydrogen bonds were observed.

In the crystal structure, the molecules are stacked along the *b* axis and the 'double layers' of molecules lying with all their bromomethyl groups together (Fig.2).

### Experimental

4-methoxyphenol (18.6 g,0.15 mol) was dissolved with stirring in water (80 ml) containing sodium hydroxide (9.0 g, 0.25 mol) and TBAB (0.48 g, 0.0015 mol) and then added dropwise to excess refluxing ethylene dibromide (65 g, 0.35 mol). The reaction mixture was heated under reflux for 12 h, cooled and extracted into chloroform. The combined organic extracts were washed with water, dried over Na_2_SO_4_, filtered and evaporated to dryness to yield an oil. Fractionation under reduced pressure yielded 1-Bromo-2-(4-methoxyphenoxy)ethane as a colorless oil, then cooled to give 27.3 g white solid (78.9% yield). Crystals of (I) suitable for X-ray diffraction were obtained by slow evaporation of an ethanol solution.

### Refinement

H atoms were positioned geometrically, with O—H = 0.82 and C—H = 0.93 Å for aromatic H, and constrained to ride on their parent atoms, with *U*_iso_(H) = *xU*_eq_(C/O), where *x* = 1.2 for aromatic H and *x* = 1.5 for other H.

### Crystal data

**Table:**

C_9_H_11_BrO_2_	*F*(000) = 464
*M**_r_* = 231.09	*D*_x_ = 1.617 Mg m^−^^3^
Monoclinic, *P*2_1_/*c*	Mo *K*α radiation, λ = 0.71073 Å
Hall symbol: -P 2ybc	Cell parameters from 25 reflections
*a* = 21.112 (4) Å	θ = 9–13°
*b* = 5.4180 (11) Å	µ = 4.29 mm^−^^1^
*c* = 8.3230 (17) Å	*T* = 293 K
β = 94.54 (3)°	Block, colourless
*V* = 949.0 (3) Å^3^	0.20 × 0.10 × 0.10 mm
*Z* = 4	

### Data collection

**Table:**

Enraf–Nonius CAD-4 diffractometer	1050 reflections with *I* > 2σ(*I*)
Radiation source: fine-focus sealed tube	*R*_int_ = 0.073
graphite	θ_max_ = 25.3°, θ_min_ = 1.9°
ω/2θ scans	*h* = −25→0
Absorption correction: ψ scan (North *et al.*, 1968)	*k* = 0→6
*T*_min_ = 0.481, *T*_max_ = 0.674	*l* = −9→9
1759 measured reflections	3 standard reflections every 200 reflections
1713 independent reflections	intensity decay: 1%

### Refinement

**Table:**

Refinement on *F*^2^	Secondary atom site location: difference Fourier map
Least-squares matrix: full	Hydrogen site location: inferred from neighbouring sites
*R*[*F*^2^ > 2σ(*F*^2^)] = 0.069	H-atom parameters constrained
*wR*(*F*^2^) = 0.155	*w* = 1/[σ^2^(*F*_o_^2^) + (0.060*P*)^2^ + 5.*P*] where *P* = (*F*_o_^2^ + 2*F*_c_^2^)/3
*S* = 1.01	(Δ/σ)_max_ < 0.001
1713 reflections	Δρ_max_ = 0.66 e Å^−^^3^
110 parameters	Δρ_min_ = −0.55 e Å^−^^3^
0 restraints	Extinction correction: *SHELXL97* (Sheldrick, 2008), Fc^*^=kFc[1+0.001xFc^2^λ^3^/sin(2θ)]^-1/4^
Primary atom site location: structure-invariant direct methods	Extinction coefficient: 0.010 (2)

### Special details

**Table:**

Geometry. All e.s.d.'s (except the e.s.d. in the dihedral angle between two l.s. planes) are estimated using the full covariance matrix. The cell e.s.d.'s are taken into account individually in the estimation of e.s.d.'s in distances, angles and torsion angles; correlations between e.s.d.'s in cell parameters are only used when they are defined by crystal symmetry. An approximate (isotropic) treatment of cell e.s.d.'s is used for estimating e.s.d.'s involving l.s. planes.
Refinement. Refinement of *F*^2^ against ALL reflections. The weighted *R*-factor *wR* and goodness of fit *S* are based on *F*^2^, conventional *R*-factors *R* are based on *F*, with *F* set to zero for negative *F*^2^. The threshold expression of *F*^2^ > σ(*F*^2^) is used only for calculating *R*-factors(gt) *etc*. and is not relevant to the choice of reflections for refinement. *R*-factors based on *F*^2^ are statistically about twice as large as those based on *F*, and *R*- factors based on ALL data will be even larger.

### Fractional atomic coordinates and isotropic or equivalent isotropic displacement parameters (Å^2^)

**Table:**

	*x*	*y*	*z*	*U*_iso_*/*U*_eq_	
Br	0.43028 (5)	0.39656 (16)	0.68335 (11)	0.0565 (4)	
O1	0.0725 (3)	0.4636 (12)	0.1822 (7)	0.0603 (17)	
C1	0.0493 (5)	0.2506 (19)	0.0971 (11)	0.068 (3)	
H1A	0.0047	0.2689	0.0682	0.102*	
H1B	0.0564	0.1077	0.1643	0.102*	
H1C	0.0713	0.2313	0.0012	0.102*	
O2	0.3246 (3)	0.5406 (10)	0.4019 (5)	0.0443 (14)	
C2	0.1538 (4)	0.6649 (13)	0.3366 (8)	0.0344 (18)	
H2A	0.1238	0.7785	0.3664	0.041*	
C3	0.1353 (3)	0.4740 (14)	0.2328 (8)	0.0305 (17)	
C4	0.2150 (3)	0.6877 (13)	0.3951 (8)	0.0317 (17)	
H4A	0.2263	0.8141	0.4673	0.038*	
C5	0.2602 (3)	0.5311 (12)	0.3510 (7)	0.0266 (16)	
C6	0.2427 (4)	0.3359 (14)	0.2494 (8)	0.0374 (19)	
H6A	0.2731	0.2239	0.2203	0.045*	
C7	0.1801 (4)	0.3076 (14)	0.1914 (8)	0.0361 (19)	
H7A	0.1683	0.1755	0.1243	0.043*	
C8	0.3458 (4)	0.7249 (14)	0.5127 (8)	0.039 (2)	
H8A	0.3222	0.7152	0.6079	0.047*	
H8B	0.3387	0.8866	0.4646	0.047*	
C9	0.4136 (4)	0.6893 (15)	0.5575 (9)	0.0408 (19)	
H9A	0.4299	0.8313	0.6186	0.049*	
H9B	0.4360	0.6791	0.4603	0.049*	

### Atomic displacement parameters (Å^2^)

**Table:**

	*U*^11^	*U*^22^	*U*^33^	*U*^12^	*U*^13^	*U*^23^
Br	0.0742 (7)	0.0413 (5)	0.0505 (6)	0.0066 (5)	−0.0173 (4)	0.0053 (5)
O1	0.051 (4)	0.066 (4)	0.062 (4)	0.002 (3)	−0.011 (3)	−0.013 (3)
C1	0.076 (7)	0.069 (7)	0.056 (6)	−0.010 (6)	−0.021 (5)	−0.011 (5)
O2	0.061 (4)	0.051 (4)	0.019 (3)	0.002 (3)	−0.006 (2)	−0.007 (2)
C2	0.053 (5)	0.026 (4)	0.025 (4)	0.006 (3)	0.006 (3)	0.005 (3)
C3	0.036 (4)	0.037 (4)	0.018 (4)	0.008 (4)	0.000 (3)	−0.007 (3)
C4	0.045 (4)	0.022 (4)	0.027 (4)	0.002 (4)	0.001 (3)	−0.009 (3)
C5	0.046 (4)	0.023 (4)	0.010 (3)	0.002 (3)	−0.001 (3)	0.005 (3)
C6	0.058 (5)	0.033 (5)	0.020 (4)	0.004 (4)	−0.003 (3)	0.000 (3)
C7	0.064 (5)	0.026 (4)	0.016 (4)	−0.001 (4)	−0.011 (3)	−0.003 (3)
C8	0.068 (6)	0.029 (4)	0.018 (4)	−0.011 (4)	−0.008 (3)	0.005 (3)
C9	0.048 (5)	0.040 (5)	0.034 (4)	−0.016 (4)	0.000 (4)	0.004 (4)

### Geometric parameters (Å, °)

**Table:**

Br—C9	1.918 (8)	C4—C5	1.350 (9)
O1—C3	1.360 (9)	C4—H4A	0.9300
O1—C1	1.421 (11)	C5—C6	1.386 (9)
C1—H1A	0.9600	C6—C7	1.380 (10)
C1—H1B	0.9600	C6—H6A	0.9300
C1—H1C	0.9600	C7—H7A	0.9300
O2—C5	1.393 (8)	C8—C9	1.463 (10)
O2—C8	1.408 (9)	C8—H8A	0.9700
C2—C4	1.350 (10)	C8—H8B	0.9700
C2—C3	1.384 (10)	C9—H9A	0.9700
C2—H2A	0.9300	C9—H9B	0.9700
C3—C7	1.370 (10)		
			
C3—O1—C1	118.4 (7)	C6—C5—O2	114.9 (6)
O1—C1—H1A	109.5	C7—C6—C5	120.0 (7)
O1—C1—H1B	109.5	C7—C6—H6A	120.0
H1A—C1—H1B	109.5	C5—C6—H6A	120.0
O1—C1—H1C	109.5	C3—C7—C6	120.0 (7)
H1A—C1—H1C	109.5	C3—C7—H7A	120.0
H1B—C1—H1C	109.5	C6—C7—H7A	120.0
C5—O2—C8	118.5 (6)	O2—C8—C9	109.1 (7)
C4—C2—C3	120.6 (7)	O2—C8—H8A	109.9
C4—C2—H2A	119.7	C9—C8—H8A	109.9
C3—C2—H2A	119.7	O2—C8—H8B	109.9
O1—C3—C7	124.8 (6)	C9—C8—H8B	109.9
O1—C3—C2	116.4 (6)	H8A—C8—H8B	108.3
C7—C3—C2	118.8 (7)	C8—C9—Br	112.4 (5)
C2—C4—C5	121.5 (7)	C8—C9—H9A	109.1
C2—C4—H4A	119.3	Br—C9—H9A	109.1
C5—C4—H4A	119.3	C8—C9—H9B	109.1
C4—C5—C6	119.0 (7)	Br—C9—H9B	109.1
C4—C5—O2	126.1 (6)	H9A—C9—H9B	107.9
			
C1—O1—C3—C7	8.3 (11)	C8—O2—C5—C6	−176.4 (6)
C1—O1—C3—C2	−170.1 (7)	C4—C5—C6—C7	1.7 (10)
C4—C2—C3—O1	179.0 (7)	O2—C5—C6—C7	179.9 (6)
C4—C2—C3—C7	0.5 (10)	O1—C3—C7—C6	179.8 (7)
C3—C2—C4—C5	2.0 (11)	C2—C3—C7—C6	−1.8 (10)
C2—C4—C5—C6	−3.1 (10)	C5—C6—C7—C3	0.7 (10)
C2—C4—C5—O2	178.9 (6)	C5—O2—C8—C9	176.1 (6)
C8—O2—C5—C4	1.6 (9)	O2—C8—C9—Br	−68.6 (7)
